# 

**Published:** 1910-06

**Authors:** 


					CONTENTS.
Page
The Lone- Fox Lecture: The Development of the Human Skull.
By Edward Fawcett, M.D.
97
Notes on Biliary and Intestinal Sand.
By George Parker, M.D. ...
Notes on a Case of Oxycephaly.
By G. Hely-Hutchinson Almond, M.B., M.A. Oxon.
n8
Iodoform and Thyroidism.
By A. Rendle Short, M.D., B.Sc., F.R.C.S.
A Case in which a Stone formed in the Urethra around a piece
of Wood introduced into the Urethra twenty-six years before
the Removal of the Stone.
By Chas. A. Morton, F.R.C.S.
127
Bromoform Poisoning.
By Rupert Waterhouse, M.D., M.R.C.P. Lond.
129
Progress of the Medical Sciences
Medicine.
J. M. Fortesque-Brickdale, M.A., M.D.
140
Surgery.
James Swain, M.D., M.b. L?ona.
M4
Diseases of the Ear, Nose and Throat.
P. Watson Williams,
M.D. Lond.
*47
Reviews of Books
I5i
Editorial Notes
172
Notes on Preparations for the Sick
i So
The Library of the Bristol Medico-Chirurgical Society   183
Meetings of Societies
i?5
Local Medical Notes
i88

				

